# Changes in diagnostics and treatment pathways for developmental dysplasia of the hip after the introduction of national guidelines: An updated questionnaire amongst paediatric orthopaedic surgeons in The Netherlands

**DOI:** 10.1177/18632521241276367

**Published:** 2024-11-04

**Authors:** Mohamed Mai, Melinda MEH Witbreuk, Pieter Bas de Witte, Christiaan JA van Bergen

**Affiliations:** 1 Faculty of Medicine, Amsterdam UMC Location VUmc, Amsterdam, The Netherlands; 2 Department of Orthopaedic Surgery, OLVG, Amsterdam, The Netherlands; 3 Department of Orthopaedic Surgery, Leiden University Medical Center, Leiden, The Netherlands; 4 Department of Orthopaedic Surgery, Amphia Hospital, Breda, The Netherlands; 5 Department of Orthopaedic Surgery and Sports Medicine, Erasmus MC Sophia, Rotterdam, The Netherlands

**Keywords:** Developmental dysplasia of the hip, children, diagnosis, treatment, survey

## Abstract

**Purpose:**

Diagnostics and treatment pathways for developmental dysplasia of the hip are highly variable in clinical practice. Recently, two national guidelines were developed in the Netherlands, providing a uniform protocol for the diagnosis and treatment of developmental dysplasia of the hip in children under the age of 1 year. The aim of this survey study was to assess whether diagnostic and treatment strategies have changed amongst paediatric orthopaedic surgeons in the Netherlands compared to a similar survey study in 2011, after the introduction of the guidelines.

**Methods:**

A web-based online questionnaire was developed and shared amongst the members of the Dutch Paediatric Orthopaedic Society. The questions concerned the diagnosis and treatment of developmental dysplasia of the hip, ranging from mildly dysplastic to dislocated hips, in children under the age of 1 year. We used a questionnaire similar to the previous study and evaluated the results.

**Results:**

Thirty-four participants completed the survey. Regarding diagnosis and follow-up, ultrasonography was generally applied for children younger than 6 months, while radiography was more frequently used for children aged 6–12 months. In 2011, radiography was more widely applied in all age groups. Initial treatment for dysplastic, stable hips was mostly active monitoring, while this was generally a rigid splint in 2011. For dislocated unstable hips, the first step in treatment was generally the Pavlik harness, as in 2011.

**Conclusion:**

The diagnostic and treatment pathways of developmental dysplasia of the hip in children under the age of 1 year seem to have partially changed amongst Dutch paediatric orthopaedic surgeons compared to 2011, after the publication of new guidelines.

## Introduction

Developmental dysplasia of the hip (DDH) refers to pathologic development of the hip joint, which can lead to dysplasia or even hip dislocation. It is preferably diagnosed and treated at an early stage to avoid severe outcomes such as pain, walking disabilities, and/or osteoarthritis at a young age.^1^ In the Netherlands, 3%–4% of children up to 6 months of age are diagnosed with DDH, and the incidence of actual hip dislocations is 0.4%.^2^ Risk factors and tell-tale signs of DDH are,for example, breech position in the 3rd trimester, a positive family history, female gender and limited hip abduction.^3^ When diagnosed at a young age, conservative treatment is generally successful.^4^ If diagnosed at an older age and in more severe cases, more invasive treatment might be indicated, including closed or open reduction, spica casting, pelvic and/or femoral osteotomies, with more variable results.^4^

Despite the relatively high incidence and potential devastating outcomes, diagnosis and treatment pathways for DDH are highly variable in clinical practice.^5^ In a previous study of Heeres et al.^5^ a national questionnaire was applied to assess the versatile opinions and practice of paediatric orthopaedic surgeons in the Netherlands, regarding the diagnosis and treatment of DDH in children under the age of 1 year. The questionnaire was sent to all members of the Dutch Paediatric Orthopaedic Society (DPOS). The conclusion of this article was that there was no uniformity amongst paediatric orthopaedic surgeons regarding the diagnosis and treatment of DDH in children under the age of 1 year.

In 2018, the Dutch Centre for Youth Health Care (JGZ) developed a national guideline on screening of DDH.^6^ In addition, in 2021, the Dutch Orthopaedic Association (NOV) developed a national guideline on the diagnosis and treatment on stable and unstable DDH in children under the age of 1 year,^7^ with the goal of improving evidence-based care and uniformity.

The aim of the current questionnaire study was to re-assess clinical decision-making for DDH in children under the age of 1 year amongst paediatric orthopaedic surgeons in the Netherlands, after the introduction of the new ‘JGZ’ and ‘NOV’ guidelines.

## Methods

A questionnaire survey was shared by e-mail amongst 117 members of the DPOS.

### Questionnaire

The web-based online questionnaire, made in Survio,^8^ consisted of multiple-choice questions with open fields for additional comments (Supplemental material Appendix 1). The questionnaire contained questions to assess current practice in the Netherlands, regarding diagnostics as well as treatment characteristics of DDH, ranging from mildly dysplastic to subluxated and dislocated hips in children under the age of 1 year. We applied a similar questionnaire as the one used in the study of Heeres et al.,^5^ with a few minor adjustments and additions to present-day insights. Additionally, we added questions about the awareness of the orthopaedic surgeons regarding the guidelines of the ‘JGZ’^6^ and ‘NOV’,^7^ and whether these guidelines have led to a change in their diagnosis and treatment pathways.

An invitation to the online questionnaire was sent by e-mail to all members of the DPOS through the secretary of the DPOS. In case of no reply within 6 weeks, a reminder was sent by e-mail. Furthermore, the survey was given attention during a national DPOS member meeting.

### Questionnaire items

The questions involved the choice(s) of diagnostic tests for clinical examination and imaging methods for DDH screening and follow-up (see Supplemental material A). Participants were asked to rank diagnostic tests by priority with regards to type of physical examination test, radiography and/or ultrasonography. This was irrespective of age group or type of DDH (dislocated/dysplastic hips). Also, the threshold imaging parameter values for treatment were assessed.

Additionally, the questionnaire included questions about the optimal treatment of DDH. The first-, second- and third choice treatment methods were asked for both dysplastic stable hips and subluxated/dislocated unstable hips. Moreover, the maximum duration of these treatment methods was assessed, as well as alternative treatment choices (e.g. closed or open hip reduction in case of no hip centralization). Lastly, the orthopaedic surgeons were asked about their treatment evaluation methods during follow-up.

### Statistics

Outcomes were mainly presented with descriptive data. Categorial data (diagnostic clinical examination, ultrasonography, radiography, the first-choice treatment by age and severity and the evaluation of treatment) were presented in proportions and percentages. Numerical data (mean duration of the first-choice treatment) were presented in means and ranges.

The current outcomes were visually compared to the 2011 questionnaire outcomes with the use of Microsoft Excel (MS Excel). No statistical comparisons were performed due to the relatively small number of participants and large number of questionnaire items (multiple testing).

## Results

Thirty-four members (29%) of the DPOS completed the survey. Of these respondents, 19 (56%) were employed at general hospitals, 13 (38%) at academic hospitals and two respondents (6%) were employed in both general as well as academic hospitals. Twenty-two of the respondents (65%) had a fulltime position. Overall, the mean number of experience years was 11.7 years (range, 1–30 years).

A total of 32 respondents (91%) indicated they knew about the ‘JGZ’ guideline, while all respondents were aware of the ‘NOV’ guideline. A total of 9 respondents (27%) reported that the ‘JGZ’ guideline had influenced their diagnostic and treatment pathways, while 16 (47%) reported that their diagnostic and treatment pathways were influenced by the ‘NOV’ guideline.

### Diagnostic clinical examination

All 34 respondents used physical examination as a diagnostic tool for children with DDH younger than 6 months. The Ortolani test^9^ was used by 32 (94%), the Galeazzi test^10^ by 28 (82%) and the Barlow test^9^ by 25 respondents (74%) (Table 1). A total of 21 respondents (62%) used all these three methods of physical examinations as a diagnostic measure.

**Table 1.** Use of clinical examination for diagnosis by age category.

Diagnostic clinical examination	0–6 months *N* = 34	6–12 months *N* = 34
Ortolani test	32 (94%)	17 (50%)
Galeazzi test	28 (82%)	26 (76%)
Barlow test	25 (74%)	17 (50%)

Also, for children of 6–12 months old, all respondents used physical examination for diagnostic purposes. For these children, the Ortolani test was used by 17 (50%), the Galeazzi test by 26 (76%) and the Barlow test by 17 respondents (50%) (Table 1). Hence, the Galeazzi test was overall considered as the most important clinical examination diagnostic test for children younger than 12 months.

### Ultrasonography

For children younger than 6 months, all 34 respondents used ultrasonography as diagnostic method for DDH. Graf’s classification^11,12^ was used by all respondents (Table 2). Moreover, 12 of these respondents (35%) also used the percentage of the femoral head coverage of the acetabulum.^13^

**Table 2.** Use of ultrasonography for diagnosis by age category.

Ultrasonography	0–6 months *N* = 34	6–12 months *N* = 23
Graf classification	34 (100%)	23 (100%)
The percentage of the femoral head coverage of the acetabulum	12 (35%)	13 (39%)
The thickness of the cartilage of the acetabulum	3 (9%)	2 (9%)
Three-dimensional ultrasonography	1 (3%)	1 (4%)

For the age category 6–12 months, ultrasonography was applied by 23 respondents (68%). In this group, all 23 respondents used Graf’s classification. The percentage of the femoral head coverage was used by 13 respondents (39%) (see Table 2). The maximum mean age at which the surgeons would use ultrasonography in children with DDH under the age of 1 year was 7.7 months on average (range, 3.0–12.0 months).

### Radiography

For children under the age of 6 months, radiographs were used for screening/diagnostics by 12 respondents (35%) in addition to the ultrasound evaluation that all respondents applied. For diagnostic measures, the acetabular index^14^ was used by 10 respondents (83%) and the Shenton–Menard line also by 10 respondents (83%) (Table 3). In children aged 6–12 months, radiographs were used by 30 respondents (88%). For this age range, the acetabular index was used by 29 respondents (97%) and the Shenton–Menard line by 23 respondents (77%) (Table 3).

**Table 3.** Use of radiography measures by age category.

Radiography	0–6 months *N* = 12	6–12 months *N* = 30
Acetabular index	10 (83%)	29 (97%)
Centre-edge angle	–	6 (20%)
Medial joint space	4 (33%)	16 (53%)
Shenton–Menard line	10 (83%)	23 (77%)
The migration percentage	2 (17%)	9 (30%)
Perkins line	7 (58%)	20 (67%)

### Treatment of stable DDH (Graf IIA, IIB, IIC)

With respect to stable DDH in children under the age of 6 months, active monitoring was applied as first-choice treatment by 22 respondents (65%) (Table 4). If active monitoring did not result in improvement of the hip (i.e. Graf I), a Pavlik harness was mostly used as second step in treatment by 23 respondents (68%), and overall, 12 respondents (35%) directly started with a Pavlik harness, instead of active monitoring.

**Table 4.** First-choice treatment by age and severity of DDH.

Treatment	Dysplasia <6 months	Dysplasia 6–12 months	Dislocation <6 months	Dislocation 6–12 months
Pavlik harness	12 (35%)	21 (62%)	34 (100%)	24 (73%)
Active monitoring	22 (65%)	3 (9%)	–	–
Rigid splint	–	9 (26%)	–	3 (9%)
Plaster cast*	–	–	–	–
Closed reduction	–	–	–	4 (12%)
Other	–	1 (3%)*	–	2 (6%)

* The indication for a plaster cast is either a closed or open reduction.

From the age of 6 months old and older, a Pavlik harness was predominantly used as first step in treatment by 21 respondents (62%) (Table 4). A rigid splint was mostly used as second-choice treatment by 18 of the surgeons (53%) when the Pavlik harness failed. In the Netherlands, usually a camp spreader is used as rigid splint.

### Treatment of unstable DDH (Graf D, III, IV)

Regarding unstable DDH (Graf D/III/IV) in children younger than 6 months, all 34 respondents indicated the immediate start of treatment with a Pavlik harness (Table 4). In case that the first choice Pavlik harness treatment did not result in centralization, 31 respondents (91%) used closed reduction and/or spica casting. For children of 6–12 months with Graf D/III/IV DDH, the majority (73%) also applied a Pavlik harness as the first step in treatment. (Table 4). Closed reduction was used by 53% as second-choice treatment if Pavlik harness failed as prior treatment.

Hence, closed reduction was generally applied in case of a failed abduction device in dislocated hips irrespective of the age. Overall, 25 respondents (74%) used closed reduction, and all reported that they would apply adductor tenotomy if closed reduction was hindered by restricted hip abduction. Two (6%) of these respondents used traction prior to adductor tenotomy.

Open reduction was applied by 25 respondents (74%) as the next step, in case their prior treatment failed. The direct anterior approach was applied by 64%, while the anterolateral and medial approaches were used by 16% and 13%, respectively. In addition, 7% used another approach (posterolateral).

Mean duration of treatment The maximum mean duration for first-choice treatment was the shortest (1.4 months) for the application of a Pavlik harness in children under the age of 6 months with a hip dislocation (See Supplemental material B, Table 5) and was the longest (4.5 months) for active monitoring in children of 6–12 months old with hip dysplasia (See Supplemental material B, Table 5).

### Evaluation of treatment

Ultrasonography was eminently used for the evaluation of all treatment techniques (Table 6). Most surgeons applied multiple forms of imaging techniques as evaluation tool, depending on the given treatment.

**Table 6.** The treatment evaluation.

Treatment	Ultrasonography	Radiography	CT/MRI	Arthrography	Other/no imaging
Pavlik harness	30 (88%)	14 (41%)	–	–	–
Rigid splint	15 (44%)	14 (41%)	1 (3%)	1 (3%)	–
Traction	2 (6%)	2 (6%)	–	2 (6%)	–
Plaster cast after closed reduction	19 (56%)	17 (50%)	8 (24%)	7 (21%)	–
Plaster cast after open reduction	17 (50%)*	17 (50%)	10 (29%)	(3%)	–

* Ultrasound examination of a baby in a spica cast is only possible with a transinguinal approach.^15,16^

### Current results in light of the 2011 survey

In the current survey study, ultrasound evaluation (Graf classification) was overall ranked as most important diagnostic method, in addition to physical examination, in the screening for and evaluation of DDH. This contrasts with the survey in 2011 of Heeres et al.,^5^ where the abduction test and radiographs (acetabular index) were both considered as the most important evaluation methods by 13 paediatric orthopaedic surgeons (33%), respectively.

With regards to clinical examination, the tests used were rather similar between both survey studies with only an increase in the use of the Ortolani test. Apart from the abduction test, the Galeazzi test was used the most by 29 surgeons (76%) and the Ortolani test was used by 25 surgeons (65%) in 2011. In the current study, the Ortolani test was used the most by 32 surgeons (94%) for children younger than 6 months old, while the Galeazzi test was used the most by 26 surgeons for children older than 6 months old (76%).

Concerning imaging, radiography (acetabular index according to Tönnis) was overall most frequently used by 33 surgeons (86%) in 2011. On the contrary, ultrasonography (according to Graf’s classification) was by far the most frequently used imaging method in the present study by all 34 surgeons (100%) for children younger than 6 months old. Also, it was used by all 23 surgeons that used any form of ultrasonography for children older than 6 months old.

For treatment of stable DDH, especially rigid splints were used in children younger than 6 months as the first choice by 21 surgeons (60%) in the 2011 survey. On the contrary, active monitoring was the most preferred initial treatment method in this age category in the current study by 22 surgeons (65%). In the 2011 survey, children older than 6 months old were also especially treated with a rigid splint by 25 surgeons (71%). In the current study, generally a Pavlik harness was used instead (21 surgeons (65%)).

For treatment of unstable DDH, a Pavlik harness was the first choice in children younger than 6 months by 32 surgeons (86%) in 2011 and by all 34 surgeons in the current study respectively. A Pavlik harness was also used the most in children older than 6 months old by 19 surgeons (52%) in 2011, as well as by 24 surgeons (73%) in the current study.

The evaluation of conservative treatment (i.e. abduction device: rigid splint or Pavlik) was primarily done with radiography in 2011: 80% (27 surgeons). In contrast, ultrasonography was especially used in the current study: 88% (30 surgeons).

## Discussion

This survey study provides an overview of current practice in the diagnosis and treatment pathways of DDH amongst Dutch orthopaedic surgeons compared to 2011, after the introduction of 2 national guidelines on DDH. A total of 16 respondents (47%) indicated their practice was changed after the introduction of the guidelines.^6,7^ Comparing with the results of a similar survey of Heeres et al.^5^ from 2011, this was predominantly indicated by: the increase in use of ultrasonography for both screening and diagnostics, as well as evaluation of treatment; the increase in the use of active monitoring as a first step in treatment of stable DDH; and the increased use of Pavlik bracing instead of rigid splint as initial treatment of stable DDH, irrespective of age. Hence, the introduction of the guidelines coincided with changed diagnostic and treatment strategies, possibly confirming their purpose in giving up-to-date and evidence-based recommendations for caretakers and in unifying clinical practice.

Regarding screening and diagnosis, the newly released national guidelines^6,7^ recommend performing hip ultrasound in children with clinical suspicion and/or risk factors for DDH. Especially the Graf method is considered the most suitable diagnostic method, until it is no longer possible to use due to ossification of the femoral head (in which radiography is recommended as alternative). A possible consequence of these recommendations is that ultrasonography (using Graf’s classification) is now more frequently used as diagnostic tool in children younger than 6 months old (100%), compared to the survey of 2011 (78%), and even for the age group of 6–12 months, ultrasonography is still frequently applied in the current study. Hence, the results of the current study were different compared to the survey of 2011, in which especially radiography (acetabular index) was used in all children younger than 12 months old. Despite this difference, it must be noted that it is unknown whether the diagnostic accuracy of ultrasonography is comparable with radiography in children younger than 1 year.^17–19^ Nevertheless, ultrasonography in the form of Graf’s method is widely accepted in the Netherlands.^6,7^

With respect to treatment, recommendations in the guidelines^6,7^ differ between stable and unstable DDH. In the current study, stable dysplastic hips in children younger than 6 months were mostly treated in line with the guidelines,^6,7^ whereby an active monitoring policy was recommended in children up to 3 months old to prevent unnecessary treatment. From 3 months and older, active monitoring is advised, with ultrasonography every 6 weeks. According to the literature, watchful waiting probably has outcomes comparable to those of an abduction brace in children with stable DDH. For example, there is probably no difference in residual dysplasia after 1 year^20^ as well as no difference in complications in patients under 1 year old treated with either watchful waiting or an abduction brace.^21^ In case of no improvement after 6 weeks, or no normalization after 12 weeks of watchful waiting, an abduction device in the form of a Pavlik harness is recommended in the recent Dutch guidelines.

Unstable dislocated hips in children younger than 6 months old and older than 6 months were also generally treated in line with the guidelines in the current survey,^6,7^ recommending a Pavlik harness as the first step in treatment for all children younger than 12 months with unstable DDH. In case of no improvements on ultrasound after 3–4 weeks, or no stable centralization after 6–8 weeks, a closed reduction is recommended. The recommended use of Pavlik as the first treatment step in dislocated hips is, incidentally, based on clinical experience, the relatively wide availability of literature on the Pavlik compared to other devices and to reduce practice variety.^22–24^ A Pavlik harness is indicated as long as the child tolerates it well. Otherwise, it is advised to change to an alternative abduction device. This may also explain why the respondents of the current study did not frequently use a rigid splint in children younger than 6 months as well as children older than 6 months old, compared to the respondents in 2011.

The current study has multiple limitations. First, although the survey was sent to all paediatric orthopaedic surgeons in the Netherlands, the response rate^25^ of this survey study was relatively low with 34 respondents (29%), especially in comparison with the study of Heeres et al. with 38 respondents (67%). However, the populations of paediatric orthopaedic surgeons of both studies were comparable, with a nearly similar mean duration of practice (11.7 years in the current study versus 12 years in the previous study, respectively) and with the majority of surgeons with an employment in general hospitals (56% in the current study versus 63% in the previous study). Second, to ensure the comparability between our questionnaire and the questionnaire study of Heeres et al. of 2011 mostly similar questions were applied. A balance had to be found between making adjustments according to present day insights and not applying too many changes to limit the comparability with the previous study. Consequently, some questions (similar as the questions from the survey of 2011) were considered as difficult to answer by a few respondents, especially with present time insights.

An example of the latter is the stratification in age subgroups of 0–6 and 6–12 months in some of the survey questions, where we now know that clinical decision-making and their outcomes might be even more age-related then suspected in 2011. The latter would justify stratification in for example: <4 weeks, 4–12 weeks, 3–6 months, 6–12 months. However, we chose to adhere to the similar methodology as in 2011 to facilitate comparisons in clinical decision-making over time. Also, in the article of Heeres et al. as well as in the current study, ultrasound was reduced to the static (Graf’s) method, while dynamic ultrasound was neglected. Dynamic examination (Harcke’s method) may have an added advantage of testing hips for subluxation or dislocation. However, there is no golden standard or reference method in case of comparing the Graf method with other ultrasonography methods. In addition, the Graf’s method is widely accepted in the Netherlands, and there is ample expertise. Aiming to reduce practice variation, no complementary methods are recommended in the Netherlands compared to the Graf method. Consequently, ultrasound was reduced to the static Graf method in the article of Heeres et al. as well as in the current article.

Lastly, we chose not to compare the 2011 and current results with statistical methods, because of variable and small populations of respondents, many outcomes (i.e. survey questions) and slight adjustments in some questions of the current questionnaire with respect to the 2011 survey.

In conclusion, the diagnostic and treatment pathways of DDH in children under the age of 1 year seem to have partly altered amongst the Dutch orthopaedic surgeons since the publication of the ‘NOV’ and ‘JGZ’ guidelines. However, this does not necessarily mean that there is causality between the changes in DDH diagnostics and treatment and these guidelines. Regarding diagnosis and follow-up, ultrasonography is more frequently used for children younger than 6 months, whilst in 2011, radiography was more widely applied. Moreover, with respect to the initial treatment, dysplastic, stable hips were often actively monitored instead of the use of a rigid splint for children younger than 6 months, as was done in 2011. However, unstable dislocated hips were often treated with a Pavlik harness, which was in line with the practice in 2011.

## Supplemental Material

sj-docx-2-cho-10.1177_18632521241276367 – Supplemental material for Changes in diagnostics and treatment pathways for developmental dysplasia of the hip after the introduction of national guidelines: An updated questionnaire amongst paediatric orthopaedic surgeons in The Netherlands Supplemental material, sj-docx-2-cho-10.1177_18632521241276367 for Changes in diagnostics and treatment pathways for developmental dysplasia of the hip after the introduction of national guidelines: An updated questionnaire amongst paediatric orthopaedic surgeons in The Netherlands by Mohamed Mai, Melinda MEH Witbreuk, Pieter Bas de Witte and Christiaan JA van Bergen in Journal of Children’s Orthopaedics

sj-docx-3-cho-10.1177_18632521241276367 – Supplemental material for Changes in diagnostics and treatment pathways for developmental dysplasia of the hip after the introduction of national guidelines: An updated questionnaire amongst paediatric orthopaedic surgeons in The Netherlands Supplemental material, sj-docx-3-cho-10.1177_18632521241276367 for Changes in diagnostics and treatment pathways for developmental dysplasia of the hip after the introduction of national guidelines: An updated questionnaire amongst paediatric orthopaedic surgeons in The Netherlands by Mohamed Mai, Melinda MEH Witbreuk, Pieter Bas de Witte and Christiaan JA van Bergen in Journal of Children’s Orthopaedics

sj-pdf-1-cho-10.1177_18632521241276367 – Supplemental material for Changes in diagnostics and treatment pathways for developmental dysplasia of the hip after the introduction of national guidelines: An updated questionnaire amongst paediatric orthopaedic surgeons in The Netherlands Supplemental material, sj-pdf-1-cho-10.1177_18632521241276367 for Changes in diagnostics and treatment pathways for developmental dysplasia of the hip after the introduction of national guidelines: An updated questionnaire amongst paediatric orthopaedic surgeons in The Netherlands by Mohamed Mai, Melinda MEH Witbreuk, Pieter Bas de Witte and Christiaan JA van Bergen in Journal of Children’s Orthopaedics
