# Supplementary material for: Changes in diagnostics and treatment pathways for developmental dysplasia of the hip after the introduction of national guidelines: An updated questionnaire amongst paediatric orthopaedic surgeons in The Netherlands
Source: J Child Orthop. 2024 Nov 4;18(6):600–6. doi: 10.1177/18632521241276367 (PMC11556652; doi:10.1177/18632521241276367)
Supplement: sj-docx-2-cho-10.1177_18632521241276367 – Supplemental material for Changes in diagnostics and treatment pathways for developmental dysplasia of the hip after the introduction of national guidelines: An updated questionnaire amongst paediatric orthopaedic surgeons in The Netherlands [file sj-docx-2-cho-10.1177_18632521241276367.docx]

| **1.** | Welke diagnostische methode gebruikt u voor DDH bij een kind < 6 maanden? | | | | |
| --- | --- | --- | --- | --- | --- |
|  | **Lichamelijk onderzoek** | | **Echografie** | | Röntgen |
|  | Barlow test  Ortolani test  Galeazzi-test |  | Graf’s α/β-hoek  Percentage acetabulum bedekking van femurkop  Dikte kraakbeen van het acetabulum  Driedimensionale echografie |  | AC-hoek  CE-hoek  Shenton-Menard-lijn  Medial joint space  Migratie percentage  Perkins/ Ombrédanne quadranten |
|  | Welke diagnostische methode gebruikt u voor DDH bij een kind van 6-12 maanden? | | | | |
|  | **Lichamelijk onderzoek** | | **Echografie** | | Röntgen |
|  | Barlow test  Ortolani test  Galeazzi-test |  | Graf’s α/β-hoek  Percentage acetabulum bedekking van femurkop  Dikte kraakbeen van het acetabulum  Driedimensionale echografie |  | AC-hoek  CE-hoek  Shenton-Menard-lijn  Medial joint space  Migratie percentage  Perkins/ Ombrédanne quadranten |

| **2.** | **Uw diagnose is gebaseerd op verschillende diagnostische methoden. Geef hier aan welke methode (van lichamelijk onderzoek/echografie/röntgen) u het belangrijkst vindt. Bijvoorbeeld 1. Graf’s α/β-hoek** | | |
| --- | --- | --- | --- |
| 1)  2)  3) | |  |  |
|  |  |  |  |
|  |  |  |  |

| **3.** | **Vanaf welk type spreekt u van te behandelen DDH bij echografie?** |
| --- | --- |


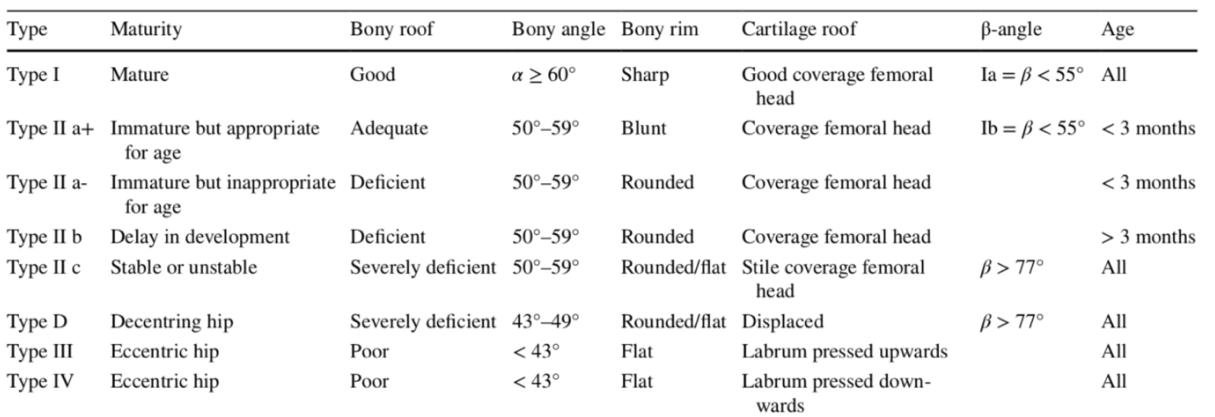

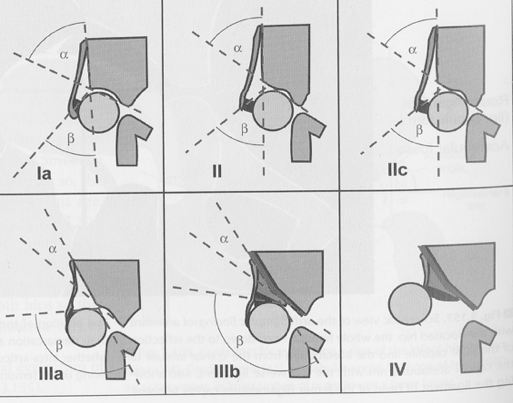


| **DDH** | **Leeftijd kind** | **I** | **IIa+** | **IIa-** | **IIb** | **IIc** | **D** | **III** | **IV** |
| --- | --- | --- | --- | --- | --- | --- | --- | --- | --- |
|  | 0-3 maanden |  |  |  |  |  |  |  |  |
|  | 3-6 maanden |  |  |  |  |  |  |  |  |
|  | 6-12 maanden |  |  |  |  |  |  |  |  |

| **4.** | **Tot en met welke leeftijd maakt u gebruik van echografie in weken?** |
| --- | --- |
|  |  |

| **5.** | **Vanaf hoeveel graden in de AC-hoek bij röntgengrafie spreekt u van te behandelen DDH?** |
| --- | --- |
|  | |
| **Tönnis tabel** | |
| 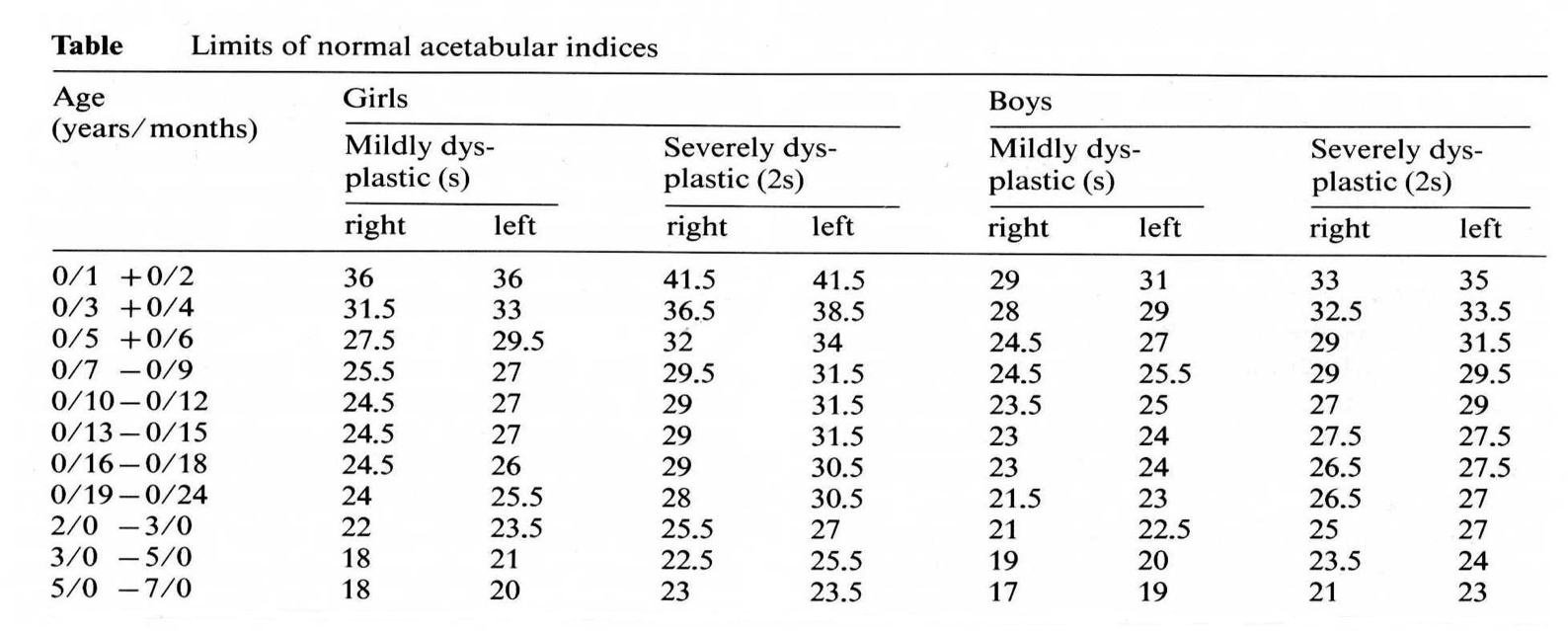 | |

| DDH rechts | **Aantal graden:** | | | |  | | | | | | | | | | | | | | | |
| --- | --- | --- | --- | --- | --- | --- | --- | --- | --- | --- | --- | --- | --- | --- | --- | --- | --- | --- | --- | --- |
| **Leeftijd kind** | **40** | **39** | **38** | **37** | **36** | **35** | **34** | **33** | **32** | **31** | **30** | **29** | **28** | **27** | **26** | **25** | **24** | **23** | **22** | 21 |
| 3-6 maanden |  |  |  |  |  |  |  |  |  |  |  |  |  |  |  |  |  |  |  |  |
| 6-12 maanden |  |  |  |  |  |  |  |  |  |  |  |  |  |  |  |  |  |  |  |  |
| DDH links | **Aantal graden:** | | | |  | | | | | | | | | | | | | | | |
| **Leeftijd kind** | **40** | **39** | **38** | **37** | **36** | **35** | **34** | **33** | **32** | **31** | **30** | **29** | **28** | **27** | **26** | **25** | **24** | **23** | **22** | 21 |
| 3-6 maanden |  |  |  |  |  |  |  |  |  |  |  |  |  |  |  |  |  |  |  |  |
| 6-12 maanden |  |  |  |  |  |  |  |  |  |  |  |  |  |  |  |  |  |  |  |  |

| **6.** | **Wanneer start u met de behandeling van stabiele DDH (IIa, IIb, IIc)?** | |
| --- | --- | --- |
|  | Meteen, maar bij een kind < 4 weken, eerst ontwikkeling volgen, dan zonodig behandelen  Meteen, maar bij een kind < 6 weken, eerst ontwikkeling volgen, dan zonodig behandelen  Meteen, maar bij een kind < 2 maanden, eerst ontwikkeling volgen, dan zonodig behandelen  Meteen, maar bij een kind < 3 maanden, eerst ontwikkeling volgen, dan zonodig behandelen  Altijd meteen behandelen | |
| Aanvulling: | |  |

|  | **Wanneer start u met de behandeling van instabiele DDH (D, III, IV)?** | |
| --- | --- | --- |
|  | Meteen, maar bij een kind < 4 weken, eerst ontwikkeling volgen, dan zonodig behandelen  Meteen, maar bij een kind < 6 weken, eerst ontwikkeling volgen, dan zonodig behandelen  Meteen, maar bij een kind < 2 maanden, eerst ontwikkeling volgen, dan zonodig behandelen  Meteen, maar bij een kind < 3 maanden, eerst ontwikkeling volgen, dan zonodig behandelen  Altijd meteen behandelen | |
| Aanvulling: | |  |

| **7.** | **1)**  **2)**  **3)** | **Met welke behandeling begint u bij een kind < 6 maanden? (vul in onder 1) 🡪 vul daarna in: voor maximaal hoelang, bij geen succes, u behandelt.** | | | | | | | | | | |
| --- | --- | --- | --- | --- | --- | --- | --- | --- | --- | --- | --- | --- |
|  |  | **Welke behandeling(en) kiest u als de 1^e^ keus niet aanslaat? (vul in onder 2) 🡪 voor maximaal hoelang behandelt u dan?** | | | | | | | | | | |
|  |  | **Welke behandeling(en) kiest u als de 2^e^ keus niet aanslaat (vul in onder 3) 🡪 voor hoelang behandelt u dan?** | | | | | | | | | | |
| Stabiele DDH ↓1 2 3↓ 🡪 voor hoelang?↓ | | | | | | | | | | | | |
|  | | |  | Pavlik | |  | | weken | | | | |
|  |  |  |  | Actieve monitoring | |  | | weken | | | | |
|  |  |  |  | Rigide spreidbroek | |  | | weken | | | | |
|  |  |  |  | Tractie | |  | | weken | | | | |
|  |  |  |  | Gipsbroek |  | | | | weken | | | |
|  |  |  |  | Anders, namelijk: |  | | | | | | | |
| Instabiele DDH↓1 2 3↓ 🡪 voor hoelang?↓ | | | | | | | | | | | | |
| Aanvulling: | | |  | Pavlik | |  | | weken | | | | |
|  |  |  |  | Rigide spreidbroek | |  | | weken | | | | |
|  |  |  |  | Tractie | |  | | weken | | | | |
|  |  |  |  | Onbloedige repositie | | Bij welke leeftijd reponeert u? vanaf | | | | |  | weken |
|  |  |  | Bloedige repositie | | |  | Med benadering | | | vanaf |  | weken |
|  |  |  |  |  |  |  | Ant-lat benadering | | | vanaf |  | weken |
|  |  |  |  |  |  |  | Ant benadering | | | vanaf |  | weken |
|  |  |  |  |  |  |  | Overig: | | | vanaf |  | weken |
|  |  |  |  | Gipsbehandeling na repositie | |  | | weken | | | | |
|  |  |  |  | Anders, namelijk: |  | | | | | | | |
|  |  |  |  | | | | | | | | | |

| **8.** | **1)**  **2)**  **3)** | **Met welke behandeling begint u bij een kind van 6 - 12 maanden? (vul in onder 1) 🡪 vul daarna in: voor hoelang bij geen succes, of tot welke leeftijd van het kind, u behandelt.** | | | | | | | | | | | | | | |
| --- | --- | --- | --- | --- | --- | --- | --- | --- | --- | --- | --- | --- | --- | --- | --- | --- |
|  |  | **Welke behandeling(en) kiest u als de 1^e^ keus niet aanslaat ? (vul in onder 2) 🡪 voor maximaal hoelang, of tot welke leeftijd, behandelt u dan?** | | | | | | | | | | | | | | |
|  |  | **Welke behandeling(en) kiest u als de 2^e^ keus niet aanslaat? (vul in onder 3) 🡪 voor hoelang behandelt u dan?** | | | | | | | | | | | | | | |
| **Stabiele DDH ↓1 2 3↓ 🡪 voor hoelang?↓ tot welke leeftijd?↓** | | | | | | | | | | | | | | | | |
|  | | |  | | Pavlik |  | | | weken |  | | weken | | | | |
|  |  |  |  | | Actieve monitoring |  | | | weken |  | | weken | | | | |
|  |  |  |  | | Rigide spreidbroek |  | | | weken |  | | weken | | | | |
|  |  |  |  | | Tractie |  | | | weken |  | | weken | | | | |
|  |  |  |  | | Gipsbehandeling |  | | | weken | |  | weken | | | | |
|  |  |  |  | | Anders, namelijk: |  | | | | | | | | | | |
| **Instabiele DDH ↓1 2 3↓ 🡪 voor hoelang?↓ tot welke leeftijd?↓** | | | | | | | | | | | | | | | | |
|  | | |  | | Pavlik |  | | | weken | |  | | | weken | | |
|  |  |  |  | | Rigide spreidbroek |  | | | weken | |  | | | weken | | |
|  |  |  |  | | Tractie |  | | | weken | |  | | | weken | | |
|  |  |  |  | | Onbloedige repositie | Bij welke leeftijd reponeert u? vanaf | | | | | | | | |  | weken |
|  |  |  | Bloedige repositie | | |  | | Med benadering | | | | | vanaf | |  | weken |
|  |  |  |  |  |  |  | | Ant-lat benadering | | | | | vanaf | |  | weken |
|  |  |  |  | | |  | | Ant benadering | | | | | vanaf | |  | weken |
|  |  |  |  | | |  | | Overig: | | | | | vanaf | |  | weken |
|  |  |  |  | | Gipsbehandeling na repositie |  | | | weken | | | | | | | |
|  |  |  |  | Anders, namelijk: | | |  | | | | | | | | | |
| Aanvulling: | | |  | | | | | | | | | | | | | |

| **9.** | **Indien een onbloedige repositie wordt belemmerd door een beperkte heupabductie, heeft dan tractie of een adductorentenotomie de voorkeur?** |
| --- | --- |
| Tractie | |
| Adductorentenotomie | |
| Anders: | |

| **10. Hoe controleert u of de behandeling succesvol verloopt?** | | | | | | |  |
| --- | --- | --- | --- | --- | --- | --- | --- |
|  | | Echografie | | Röntgen | CT/MRI | Arthrogram | Anders/zonder beeldvorming |
| Pavlik | |  | |  |  |  |  |
| Rigide spreidbroek | |  | |  |  |  |  |
| Tractie | |  |  | |  |  |  |
| Onbloedige repositie in gipsbroek | |  |  | |  |  |  |
| Bloedige repositie in gipsbroek | |  |  | |  |  |  |
| Aanvulling: |  | | | | | |  |

| **11.** | **Bent u bekend met de richtlijnen van de JGZ (2018) en NOV (2020) omtrent de diagnostiek en behandeling van DDH bij kinderen < 12 maanden?** | | Ja | Nee |
| --- | --- | --- | --- | --- |
|  | **Zo ja: hebben de DDH richtlijnen van de JGZ (2018) en/of NOV (2020) uw diagnostiek en keuze van behandeling omtrent DDH bij kinderen < 12 maanden veranderd, en op welke manier?** | | Ja | Nee |
| Aanvulling: | |  | | |

| **12.** | **In wat voor ziekenhuis werkt u?** | | | **perifeer** | | **academisch** |
| --- | --- | --- | --- | --- | --- | --- |
|  | | | | | | |
|  | **Hoeveel jaar ervaring heeft u?** | |  | | **jaar** | |
|  | | | | | | |
|  | **Hoeveel werkt u?** | **fulltime** | | **parttime Hoeveel fte?** | | |
|  | | | | | | |
|  | **Bent u WKO-lid?** | **Ja** | | **Nee** | | |
|  | | | | | | |
|  | **Welk aandeel van uw patiënten valt onder de kinderorthopedie (%)?** | **…%** | | | | |

| **13.** | **Heeft u nog aanvullingen over de diagnose of behandeling van DDH?** |
| --- | --- |
|  | |
