# Supplementary material for: Changes in diagnostics and treatment pathways for developmental dysplasia of the hip after the introduction of national guidelines: An updated questionnaire amongst paediatric orthopaedic surgeons in The Netherlands
Source: J Child Orthop. 2024 Nov 4;18(6):600–6. doi: 10.1177/18632521241276367 (PMC11556652; doi:10.1177/18632521241276367)
Supplement: sj-docx-3-cho-10.1177_18632521241276367 – Supplemental material for Changes in diagnostics and treatment pathways for developmental dysplasia of the hip after the introduction of national guidelines: An updated questionnaire amongst paediatric orthopaedic surgeons in The Netherlands [file sj-docx-3-cho-10.1177_18632521241276367.docx]

**Table 5.** The maximum mean duration for first-choice treatment

| **Treatment Dysplasia Dysplasia Dislocation Dislocation**  **<6 months 6-12 months < 6 months 6-12 months** | | | | |
| --- | --- | --- | --- | --- |
| Pavlik harness | 3.8 (0.5-10.0) | 2.8 (0.5-6.5) | 1.4 (0.5-3.0) | 1.6 (0.5-4.5) |
| Active monitoring | 1.7 (0.5-3.0) | 4.5 (3.0-6.0) | - | - |
| Rigid splint | - | 3.2 (1.0-6.0) | - | 2.2 (1.0-3.0) |
| Plaster cast | - | - | - | - |
| Closed reduction | - | - | - | 3.0 (-) |

*Numbers represent means (months) with ranges between brackets*
